# Does Drinking Coffee Reduce the Incidence of Parkinson's Disease?

**DOI:** 10.7759/cureus.34296

**Published:** 2023-01-27

**Authors:** Tsz Ki Ko

**Affiliations:** 1 Otolaryngology, College of Life Sciences, Leicester Medical School, George Davies Centre, Leicester, GBR

**Keywords:** neurology and neurosurgery, levodopa-carbidopa, caffeine treatment, coffee consumption, parkinson' s disease

## Abstract

Parkinson’s disease (PD) is an increasing threat to first-world nations as their population ages, with around one in 100 suffering from it by age 60. Incurable, with treatments that do little to delay disease progression, PD induces severe disability and even death in those afflicted. The search for preventative measures has revealed the widely used psychoactive stimulant caffeine, which competitively inhibits adenosine receptors to induce a wide variety of effects. The inhibition of inflammation and microglial cell activation to reduce reactive oxygen species (ROS)-induced cellular damage and the resultant mitochondrial dysfunction of the dopaminergic neurons appears to be the main pathway, inducing neuronal loss via the activation of the intrinsic pathway to apoptosis.

Mouse models and human data reinforce that caffeine delays the onset of PD in a dose-dependent manner. Evidence suggests it is more beneficial in men than women and is not beneficial at all in women undergoing hormone replacement therapy (HRT). Additionally, some studies suggest that although caffeinated drinks such as cola and tea are beneficial, there may be other products in coffee that prevent the effect, though this requires further research.

Although there is strong evidence that caffeine is neuroprotective, there is less evidence that it delays the onset of PD. Given the association with cardiovascular disease, it may be disadvantageous overall to the majority of the population to supplement caffeine, though still a beneficial preventative technique for individuals with a genetic predisposition to PD that may otherwise suffer early onset.

## Introduction and background

Parkinson or Parkinson's disease (PD) was first described as a single disease in 1817 by James Parkinson, but it was another century before the disease was linked to neuronal loss in the substantia nigra pars compacta (SNpc). After dopamine (DA) loss was discovered in the post-mortem brains of Parkinson's patients in 1958 by Arvid Carlsson, it was found that the SNpc played an essential role in the nigrostriatal dopaminergic pathway. Thus, SNpc degeneration led to DA deficiency, and the replenishment of DA via oral consumption of the DA precursor levodopa (L-3,4-dihydroxyphenylalanine, L-DOPA) resulted in alleviated symptoms [1].

Both chronic and progressive, the mean onset of PD is at roughly 55 years of age, with many cases resulting in total physical incapacitation within 10 years of onset [2]. Eighty percent of patients have idiopathic PD with no known cause, and ninety-five of cases are sporadic with no genetic predisposition [2,3]. Much of the difference is the result of secondary Parkinsonism, which results from other diseases such as viral infections, brain tumours, and encephalitis, or from the actions of neuroleptic drugs and neurotoxins [2].

The mortality rate of the disease was three times that of unaffected age-matched individuals, and although this was reduced with the administration of L-DOPA, it was not reduced to control levels [1]. Additionally, treatment of rats sufficient to restore the striatal levels of dopamine caused an overflow in other brain regions, such as the hippocampus, sufficient to induce aberrant signaling [4].

Symptoms often begin with limb tremors, which progress to the whole body, including the jaw and tongue, affecting speech as well as movement. The other main symptoms are rigidity and bradykinesia/akinesia. Patients' movement is slowed (bradykinesia) or even precluded (akinesia), resulting in drooling, lack of facial expression, and reduced vocal amplitude, amongst others, with everyday tasks becoming extended or impossible.

Dementia occurs in approximately 10-20% of PD patients, which is roughly double the risks in the overall population [2]. Although the links are currently unclear, dementia is always associated with Lewy bodies in the nucleus basalis of Meynert, causing loss of cholinergic neurons in the hippocampus [5].

Current treatments are largely symptomatic in that they do not slow or halt the degeneration of dopaminergic nerves or heal damaged tissue. Therefore, treatments that may reduce the risk of developing PD are highly worth considering. This review specifically discusses the role of caffeine as a preventative measure with an outlook toward further research. However, firstly it is important to discuss the causal factors of PD so that the roles of caffeine in their inhibition can be more effectively discussed.

## Review

Causes of Parkinson's disease

About 90% of Parkinson's disease (PD) is 'idiopathic'. PD does not manifest until 70-80% of striatal dopamine is depleted, as prior to this, the remaining neurons compensate via the increased production of dopamine and dopamine receptors [2]. Once over this threshold, the basal ganglia subdivisions of the putamen and the caudate nucleus, which are normally densely innervated by these dopaminergic nerves, can no longer receive sufficient signal, thus resulting in the movement disorders described above. However, the pathways by which PD progresses after the loss of these dopaminergic nerves are not as relevant as the pathways that may result in the death of these nerves [2].

In 1983, it was discovered that the neurotoxin 1-methyl-4-phenyl-1,2,3,6-tetrahydropyridine (MPTP) induced specific neurodegeneration very similar to PD [6]. The lipophilic compound crossed the blood-brain barrier and was taken up by the lysosomes of astrocytes and serotonergic neurons containing monoamine oxidase B (MOA B), which then converted it into the toxic product 1-methyl-4-phenylpyridinium (MPP+) ± via oxidation. Once secreted back into the extracellular fluid, it is taken up by the dopamine (DA) transporters into dopaminergic nerves and transported into mitochondria, where it inhibits with complex I [7]. The result was the inhibition of adenosine triphosphate (ATP) synthesis, production of superoxide radicals, and multiple other radicals, including the highly reactive hydroxyl radical [8]. Importantly, the antioxidant enzyme superoxide dismutase (SOD) was found to be neuroprotective [9]. Such compounds have been shown to work via modulating oxidation as well as the cell death pathways [10,11].

Notably, changes in reactive oxygen species (ROS) levels occur hours after the administration of MPTP, indicating proximal changes that occur well before the induction of cell death [12], which must therefore be directed by other downstream pathways.

Potentially, high levels of ROS-induced DNA damage may be initially compensated for by repair mechanisms until the breaks begin to accumulate and overwhelm the repair machinery [13]. Thus, after enough time has passed, the DNA damage leads to the upregulation of tumor suppressor protein p53, which induces Bax translocation to the mitochondria, instigating the release of cytochrome c and the activation of effector caspases 9 and 3. The caspases then cleave the cellular DNA, bringing about cell death by the intrinsic pathway of apoptosis [14].

Importantly, prolonged MPTP exposure leads to the induction of SNpc dopaminergic neuron apoptosis, and Bax-null transgenic mice are resistant both to apoptosis and MPTP-induced PD [15]. Additionally, Jun amino-terminal kinases (JNK) activation is also shown to play a role in Bax translocation, and its inhibition also alleviates MPTP-induced neurotoxicity [16]. 

MPP+ also creates an inflammatory environment by the upregulation of several cytokines, including tumor necrosis factor-α (TNF-α), interleukin-6 (IL-6), and interleukin-1β (IL-1β) [17], and this may, in turn, increase the oxidative environment via activation of inducible and neuronal nitric oxide synthases (iNOS and nNOS) to produce nitric oxide (NO) [18]. Therefore, the key regulators of MPTP-induced PD appear to be oxidative stress, inflammation, and mitochondrial dysfunction, all of which are highly interlinked. 

Notably, MPTP was initially discovered to induce PD in humans poisoned by a by-product of narcotic synthesis [19]. However, since then, much of MPTP work has been done in animal models and may, therefore, not relate to humans.

**Figure 1:**
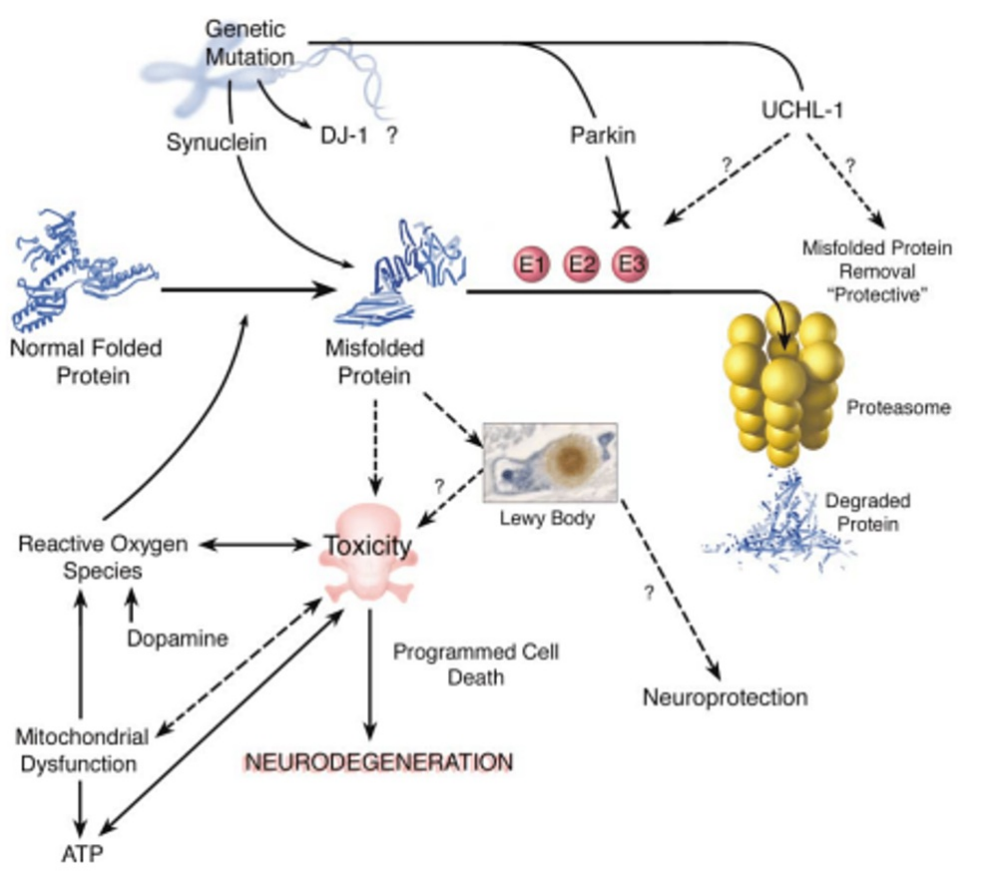
Pathways involved in the onset of PD PD - Parkinson's disease; UCHL-1  - ubiquitin C-terminal hydrolase L1; ATP - adenosine triphosphate; DJ-1 - protein deglycase (Parkinson's disease protein 7) Figure taken from [3], and permission was obtained from the original publisher to reproduce the content.

Studies in humans show that all the mutations predisposing to PD implicate the ubiquitin-proteasome pathway. One gene, α-synuclein, has two missense mutations causing dominantly inherited PD [20]. However, this does not appear to be through loss of function as, despite deficits in dopamine-dependent locomotor signaling, α-synuclein null mice were not overtly predisposed to PD [21]. As both wildtype and mutant α-synuclein form fibrils like those in Lewy bodies (Figure 1), and the mutations cause accelerated oligomerization [20], a gain of function that increases protein misfolding, aggregation, and the formation of these protofibrils is likely to explain the role of α-synuclein in PD.

One possibility is that protofibrils permeabilize synaptic vesicles, releasing dopamine into the cytoplasm, which is known to increase oxidative stress [22]. Notably, evidence suggests that in many neurodegenerative diseases, including PD, the insoluble inclusion bodies that form from protein aggregation do not contribute to cell death. Instead, the inclusions might result from an active attempt to sequester toxic soluble proteins that result from misfolding. These could induce disease directly or via inhibiting the ubiquitin-proteasome pathway, thus inhibiting protein degradation and providing positive feedback to increase the number of misfolded proteins [23]. 

Parkin, another protein mutated in early onset PD (often before age 30), is an E3 ubiquitin ligase (Figure 1), which plays an important role in protein polyubiquitination, and thus is an essential part of the protein degradation pathway by targeting them to the proteasome [24]. Indeed, several studies now suggest links between parkin and synuclein, indicating that the latter inhibits the proteasome and that mutant parkin increases sensitivity to synuclein-induced cell death [3].

Therefore, at least in humans, the main pathways by which mutations predispose to PD seem to be via the toxic effects of protein misfolding, especially on the inhibition of protein degradation by the proteasome. Importantly, despite widespread neurodegeneration, transgenic mice with α-synuclein mutations do not exhibit PD-like symptoms, and their dopaminergic neurons appear paradoxically resistant to cell death [25]. Therefore, there may be some differences in the processes involved in PD between mice and humans, but as shown in Figure 1, all the pathways are interlinked.

Protein misfolding and proteasome inhibition increase levels of ROS and are likely to affect mitochondrial function, while the aberrantly functioning mitochondria will produce more ROS and induce further protein oxidation. Notably, high ROS levels induce α-synuclein oxidation, which increases its aggregation [26], indicating another pathway by which ROS may induce PD. Equally, the lysosomal function is highly interlinked with proteasomal function, and proteasomal inhibition may contribute to reduced mitophagy [27], thus allowing dysfunctional mitochondria to persist, increasing the likelihood of cell death.

Thus, in discussing the potential effects of caffeine on PD, the key pathways to examine are protein misfolding and degradation, ROS production, and mitochondrial dysfunction.

Caffeine

Caffeine (1,3,7-trimethylxanthine) is the most widely consumed stimulant in the world. A highly lipophilic substance, it can penetrate all body tissues, including the blood-brain barrier and placenta. It does not accumulate in the body and is quickly metabolized in the liver by the isozyme cytochrome P450 1A2 (CYP1A2), part of the microsomal cytochrome P-450 system, with an average half-life of 4-6 hours and an excretion rate of 1-3% [28].

Caffeine has some effect on most organs, but it is primarily a stimulant. Fatigue is reduced, and motor activity is increased, though complex motor tasks and sleep are disrupted, potentially resulting in insomnia [28].

It also has an acute effect on blood pressure, acting as a potent vasoconstrictor, with the heart rate rising 2-3 hours after administration. While early evidence was potentially limiting effects on hypertensives [29], more recent studies suggest the effects are more robust and widespread [30]. There is also significant evidence that caffeine is associated with coronary heart disease [31], and there is evidence also of its tumorigenic properties (and anti-tumorigenic properties), depending on the dose, carcinogen, and stage of cell cycle it is introduced, although recent evidence suggests it is preventative overall [32].

Other potentially relevant effects include bronchodilation and stimulation of diuresis and lipolysis. As such, it might be expected for caffeine to have multiple mechanisms of action, but although caffeine is thought to have minimal phosphodiesterase activity and induce mobilization of calcium from the sarcoplasmic reticulum, it is mainly thought to act via antagonizing the adenosine A1 and A2a receptors (A1Rs and A2aRs, respectively) [33]. Antagonization of A1Rs and A2aRs give opposing actions: the former inhibits adenylate cyclase and activates potassium channels and phospholipases C and D while inhibiting calcium channels, while the latter activates the calcium channels. Importantly, while A1Rs are distributed widely across the brain and peripheral organs, A2Rs are localized to the nucleus accumbens, dorsal striatum, and olfactory tubercle [34], suggesting that they may mediate opposing effects in different parts of the brain.

While changes in calcium homeostasis might be primarily responsible for the vasoconstrictive and blood pressure-associated properties of caffeine, and increases in catecholamines such as adrenaline may play an important role in lipolysis, the primary effects on PD appear to come from the inhibitory effects on multiple neurotransmitters including, dopamine, glutamate, noradrenaline, acetylcholine, and γ-aminobutyric acid (GABA) [35].

The effects of caffeine on Parkinson's disease-associated pathways

GABA plays an important role in PD pathogenesis as dopamine regulates GABA signaling, and loss of dopamine results in an increase in GABA-stimulated inhibition of excitatory glutamate signaling, thus resulting in the phenotype of bradykinesia and akinesia. Importantly, adenosine is a potent stimulator of GABA signaling via the A1 and A2a receptors [35], which are known to be competitively inhibited by caffeine. However, it is noteworthy that dopamine loss is the result of the destruction of the dopaminergic neurons; thus, if this were the only effect of caffeine, it could attenuate the symptoms of PD but not the underlying cause. Similarly, adenosine signaling is potently induced by hypoxia [36] and thus may contribute to the PD phenotype in damaged and inflamed tissue. Although this could be inhibited by caffeine, the drug could not affect the inflammation and cell death that underlies it via this pathway.

Interestingly, the blood-brain barrier around the striatum was damaged in MPTP-treated mice, which were protected by the co-administration of caffeine [37]. In other areas of the brain, such as the hippocampus, the blood-brain barrier remained intact, suggesting, firstly, the effects were localized to the dopaminergic neurons and, secondly, that caffeine may inhibit PD at least partially by protecting the blood-brain barrier. This was unlikely to result from reduced vascularisation as Barcia et al. in 2005 found no evidence of increased blood vessel volume or number, although MPTP-treated monkeys did show signs of increased angiogenesis [38].

Increased permeability of the blood-brain barrier is likely to reflect high levels of inflammation and ROS. Notably, a marker of neuroinflammation is the activation of glial cells and astrocytes, known as gliosis, which is increased in PD [37]. Activated microglial cells secrete TNF-α, IL-1, ROS, reactive nitrogen species (RNS), and neurotoxic factors in an attempt to induce inflammation and clear infection [39]. Importantly, the additional microglial cells in the substantia nigra compared to other brain regions, at least in mice, might explain their sensitivity to oxidative stress and inflammation-inducing PD [40].

**Figure 2:**
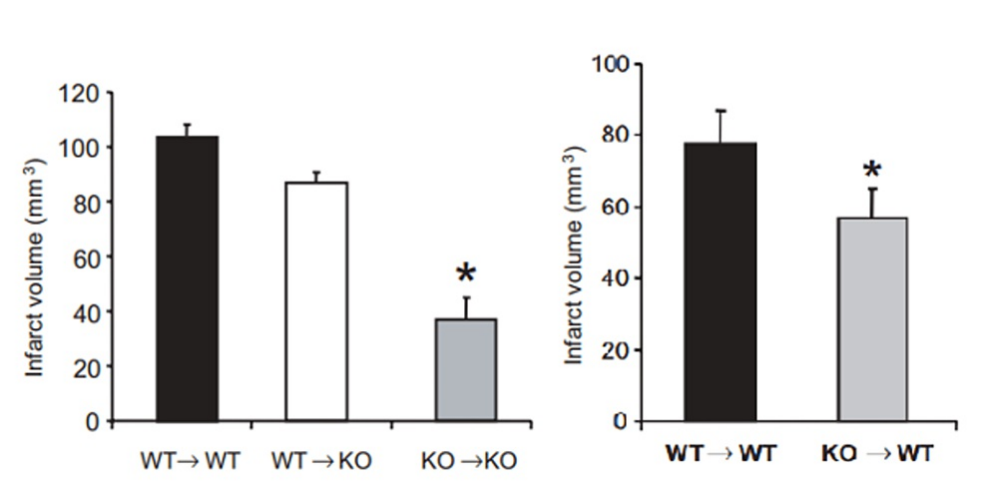
Wildtype and A2aR KO mice (WT->WT) and (KO->KO), respectively, show that (KO->KO) mice are significantly less susceptible to neural infarction induced by MPTP When the bone marrow of wildtype mice replaced the bone marrow of knockouts (WT->KO) it reduces the protective effect of the knockout to wildtype level, while replacing wildtype bone marrow with knockout (KO->WT) also significantly reduced the level of infarction. The significance of the two graphs was determined by one-way ANOVA and Student's T-test, respectively (p<0.05). A2aR - adenosine A2A receptor; KO - knock-out; WT - wild-type; MPTP - 1-methyl-4-phenyl-1,2,3,6-tetrahydropyridine Figure adapted from [41], and permission was obtained from the original publisher to reproduce the content.

A study using A2aR knockout (KO) mice showed that they were protected from the inflammatory processes involved in ischemic brain injury, suggesting that caffeine may attenuate PD via inhibition of inflammation [41]. Notably, the same study demonstrated that reconstitution of A2aR in the bone marrow in a KO background inhibited the protective effects, as shown in Figure 2.

Notably, the authors did not compare KO->WT with KO->KO mice, possibly due to insufficient animals, but comparing the graphs in Figure 2 suggests that there might still be a significant difference, and mutant bone marrow cells might not be sufficient for maximal effect.

Thus, A2aR signaling may be necessary in both cell types, and any inhibitory effect of caffeine on PD may not be limited to effects on the brain. Further complicating the issue, A2aR KO mice are susceptible to death from inflammatory signals in the peripheral organs, such as the liver and kidney, where control mice suffer only minimal damage [42], suggesting that even aside from its potential cardiac and tumorigenic properties, caffeine should be used as a preventative measure for PD with caution.

Importantly, the mechanisms of caffeine's action appear to work primarily through competitive inhibition of A2aRs, as specific inhibitors of these receptors, such as 8-(3-chlorosty-ryl)caffeine (CSC) and 3,7-dimethyl-1-propargylxanhine (DMPX) both attenuated the neurotoxic effects of MPTP, while specific interaction with A1Rs through 8-cyclopentyl-1,3-dipropylxathine (CPX) had no effect on MPTP induced neurotoxicity [43], and the same study found that A2aR KO mice had increased dopamine and dopaminergic neurons after treatment with MPTP, further suggesting that caffeine works primarily by inhibiting this receptor, at least in this animal model.

While an understanding of how caffeine may have protective qualities is crucial, there is always the possibility that these effects are counteracted by effects it has elsewhere or be of little physiological relevance. Effective assessment of the protectiveness of caffeine requires studies directly examining the outcomes of caffeine usage on PD in humans and animal models.

Animal models of caffeine and Parkinson's disease

**Figure 3:**
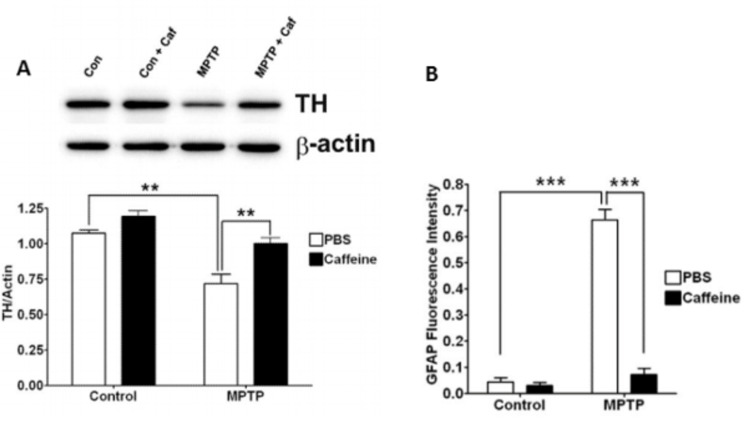
(A) shows expression of TH protein, reduced by MPTP and rescued by caffeine, with β-actin and phosphate buffer saline (PBS) used as controls. (B) shows GFAP-fluorescence increasing in response to MPTP and being rescued by caffeine. Significance was determined by one-way ANOVA with the Tukey post hoc test. Stars indicate (A) p<0.01, (B) p<0.001. TH - tyrosine hydroxylase; MPTP - 1-methyl-4-phenyl-1,2,3,6-tetrahydropyridine; GFAP - glial fibrillary acidic protein; Con - control; Caf - caffeine; PBS - phosphate-buffered saline Figure adapted from [37], and permission was obtained from the original publisher to reproduce the content.

In mice with PD induced by MPTP, co-administration of caffeine results not only in functional protection but an attenuation of dopaminergic neuronal degeneration as assessed by TH protein levels as a marker of dopaminergic function and glial fibrillary acidic protein (GFAP) as a marker of gliosis [37], as shown in Figure 3.

Although Figure 3A shows that caffeine increases TH protein in the MPTP group, it also shows a slight increase in the control group, raising the possibility that caffeine may not be inhibiting PD pathways so much as inducing compensatory mechanisms that increase dopaminergic function. Alternatively, it may reflect chance or operator bias. The complete reduction of gliosis shown in Figure 3B is more convincing and perhaps just as important, as another study found that most of the ROS in the substantia nigra resulted from the activation of inflammatory microglial cells [44].

**Figure 4:**
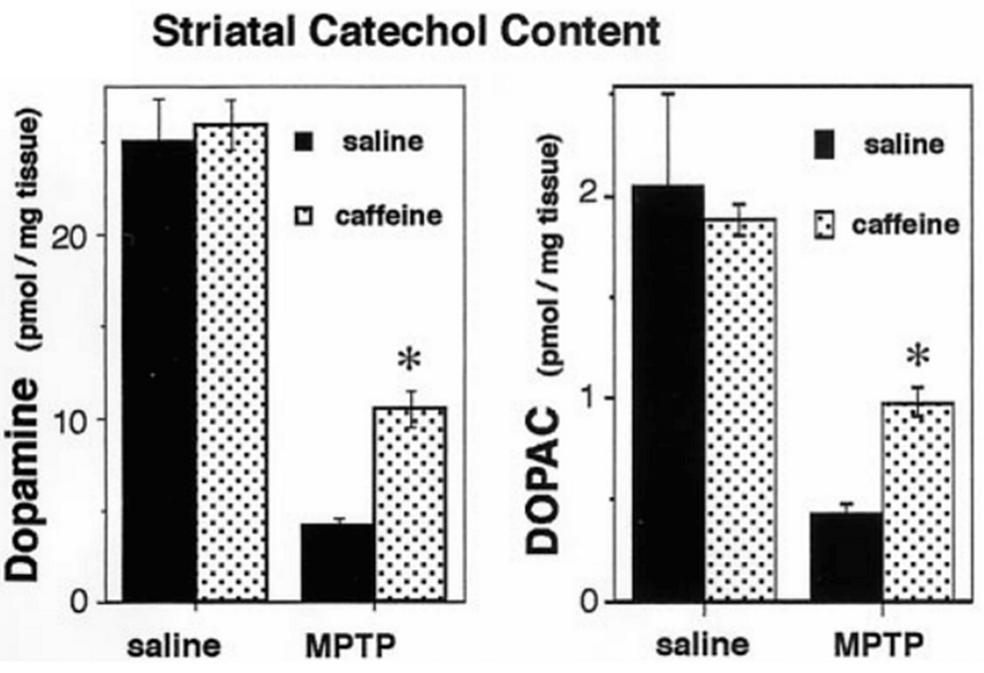
Treatment with MPTP reduces dopamine and DOPAC in the striatum of the mouse brain, which is partially rescued by caffeine Significance assessed using Student's T-test (p<0.01). MPTP - 1-methyl-4-phenyl-1,2,3,6-tetrahydropyridine; DOPAC - dihydroxyphenylacetic acid Figure adapted from [43], and permission was obtained from the original publisher to reproduce the content.

Another study investigating the same model investigated dopamine and the major metabolite of dopamine, dihydroxyphenylacetic acid (DOPAC), and got similar results, as shown in Figure 4.

The results suggest that unlike gliosis (Figure 3B), dopamine and DOPAC levels are not restored to control levels, suggesting that caffeine is not completely preventative, at least at the doses used for this experiment. Notably, both studies used the same dose of 10mg/kg, though Chen et al. (2001) used male C57BL/6 mice [43] and Chen et al. (2008) used male Friend leukemia virus B (FVB) mice [37], so the difference may be a reflection of strain genetics.

**Figure 5:**
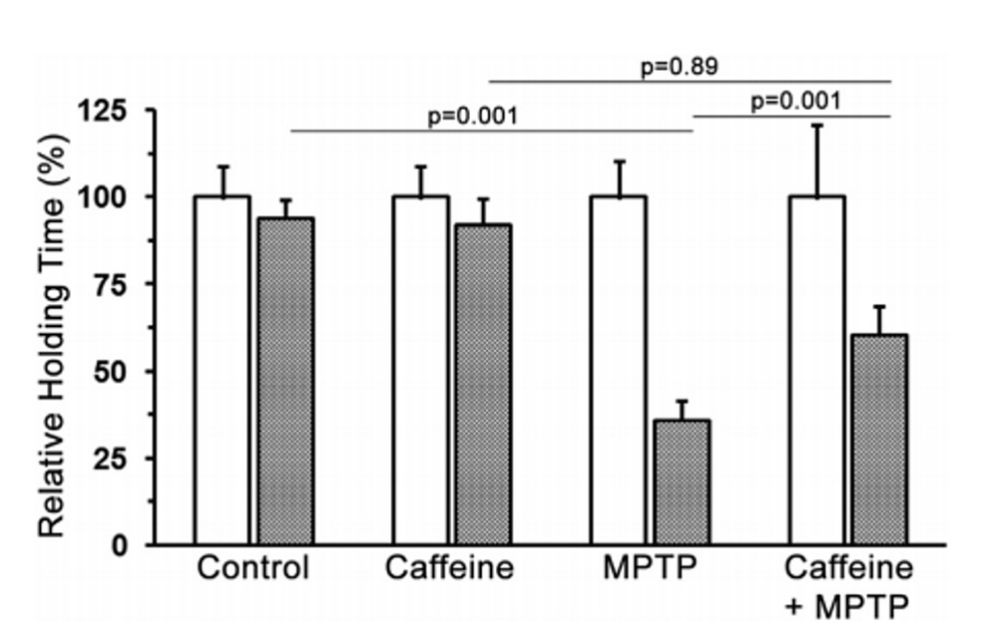
Mouse grip strength was assessed by forcing mice to hold onto a horizontal metal pole 20cm above the ground and measuring the time it took for the mice to lose grip White bars show results at zero days (pre-treatment) and grey bars show results at nine days after treatment. MPTP significantly reduces hold time, which is rescued by caffeine. Significance values shown were assessed by one-way ANOVA with post hoc Tukey honest test. MPTP - 1-methyl-4-phenyl-1,2,3,6-tetrahydropyridine Figure adapted from [45], and permission was obtained from the original publisher to reproduce the content.

Regardless, the incomplete dopamine and DOPAC restoration that resulted from pre-treatment with caffeine may potentially reflect incomplete restoration of PD symptoms. A recent study using the MPTP model assessed mouse grip strength after PD induced by MPTP treatment and rescue by caffeine [45], and the results are shown in Figure 5.

The results apparently demonstrate a complete reversal of PD-like symptoms if caffeine is administered with MPTP. However, p-values of 0.89 between caffeine and caffeine + MPTP, and 0.01 between MPTP and caffeine + MPTP, when the differences between the error bars look very similar, do arouse some suspicion as to the validity of the significance tests used. Whether or not the mice treated with caffeine + MPTP are significantly different by the ANOVA, it does appear from the plots that caffeine is not completely protective.

Notably, the low numbers used in mouse studies combined with procedural problems, including that the subjects associated with behavioral studies do not understand the purpose, and may refuse to comply, may both negatively affect the results.

Another study, subject to the same problems, showed that caffeine could at least partially reverse the effects of memory disruption in rats [46]. Although these models are highly informative as they allow a causative assessment of the role of caffeine, minimize environmental differences, and allow the use of genetically identical individuals, they suffer from the problems highlighted above, as well as from the effects of inbreeding to create strains, and their obvious difference to humans, which is emphasized by the fact that amnesia is not generally a part of human PD. To properly assess the potential for caffeine to attenuate PD in humans requires clinical and epidemiological studies.

Studies of caffeine on Parkinson's disease in humans

As early as 1968, there were found to be associations between coffee drinking and PD, which was supported by multiple studies since then [47]. In 2007, a family-based case-control study demonstrated that PD sufferers were significantly less likely to intake regular caffeine than their nonsuffering kin. Additionally, the relationship was dose-dependent with the reduced risk associated with increased consumption [48].

**Figure 6:**
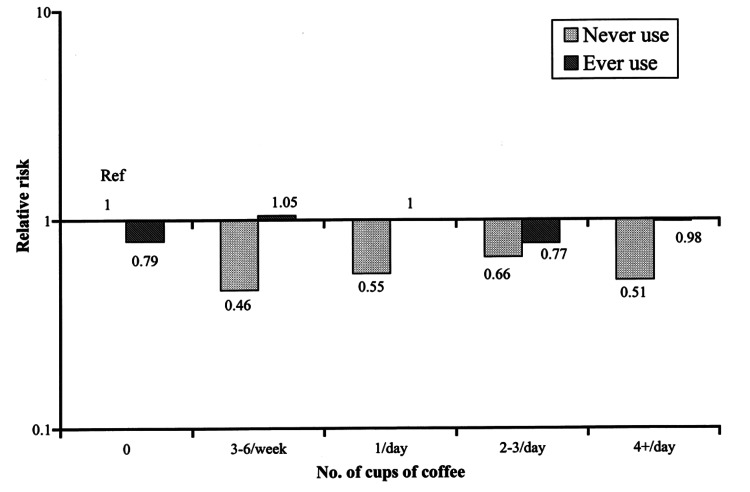
The dark grey bars (ever use) show women undergoing hormone replacement therapy (HRT), while the light grey bars (never use) are women not on HRT The results show a non-dose dependent reduction in risk of PD in the never use group only, with no significant difference in the ever use group. PD - Parkinson's disease Figure adapted from [49], and permission was obtained from the original publisher to reproduce the content.

Other studies have found the difference to be sex-dependent, with coffee consumption in men but not women associated with a lower risk of PD. The latter case seemed to be dependent on whether the women were post-menopausal and undergoing estrogen treatment [49], as shown in Figure 6.

Notably, the study by Ascherio et al. in 2004 was done from a cohort undergoing a cancer treatment study, which was why the difference in women undergoing HRT was observed, and such a group may not reflect the majority of the population [49].

Another study suggested that neither men nor women showed any significant difference in risk of developing PD with p-values of 0.063 and 0.073, respectively. Observably, these were near significant, and when both groups were combined, the significance was p=0.005 [50], suggesting that the insignificant effects in both groups alone were more of a reflection of a sample size than a lack of effect.

However, other studies have found no significant relationship between coffee consumption and PD [51], as shown in Table 1.

**Table 1:** There is no significant trend between coffee intake and the risk of PD *Odds ratios (OR) adjusted for age, ethnicity, education, and gender. CI - confidence interval; PD - Parkinson's disease Table adapted from [51], and permission was obtained from the original publisher to reproduce the content.

Coffee (regular; cups/day)	No. of cases	No. of controls	OR*	95% CI
Almost never	63	96	1.0	
>0-1	32	66	0.8	0.4 - 1.3
2-3	69	105	1.1	0.7 - 1.8
4-6	30	48	1.2	0.7 - 2.2
>6	16	32	1.0	0.5 - 2.0
p for trend				0.50

Notably, the same study [51] did find a significant trend for tea intake and a reduction from over two cups of cola per day.

Other studies also found inconsistent results, with one study demonstrating advanced age of onset of PD by 4.8 years from coffee consumption exceeding three cups per day, though again it was delayed by tea by 7.7 years from the same level of tea consumption [52]. One possibility is that caffeine is protective, while other ingredients of coffee have the opposite effect, although other studies have suggested that tea intake is less effective than coffee [50].

**Figure 7:**
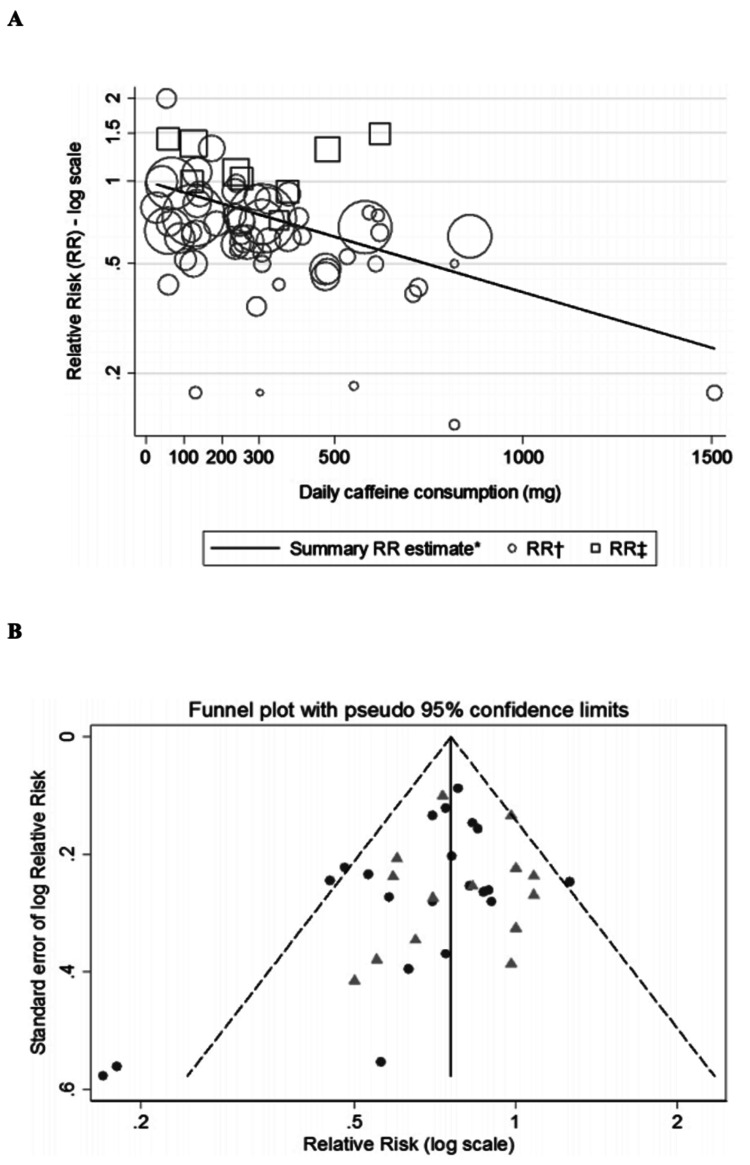
(A) shows the correlation of the risk of developing PD with increased caffeine consumption with the summary RR line representing weighted least squares regression. (B) The funnel plot demonstrates a symmetric distribution with few studies lying outside the 95% confidence interval, suggesting no significant publication bias. PD - Parkinson's disease; RR - relative risk Figure adapted from [47], and permission was obtained from the original publisher to reproduce the content.

Contrarily, a recent meta-analysis using 26 studies, although many of these did not control for smoking, showed that despite prior negative studies, the majority of the data across a wide variety of nations still suggested caffeine was protective [47], as shown in Figure 7.

Notably, there was moderate heterogeneity between studies, which was reduced from I2 = 35.1% to I2 = 27.6% if studies using women undergoing hormone replacement therapy (HRT) were removed. The association was weaker in women than in men but was consistent and robust throughout.

Caffeine and outlook to treatment

There appears to be significant evidence, reviewed above, that caffeine is protective against the onset of PD - even if it should be consumed in tea rather than coffee. However, it must also be recognized that caffeine has potentially tumorigenic properties and significantly increases the risk of heart disease [31,32]. Even discounting the former, the risk of heart disease greatly exceeds the risk of PD for the majority of the population, so consuming high doses of caffeine should be undergone with caution.

This fact is emphasized by the wealth of data that, aside from caffeine, smoking also decreases the risk of PD [47,52], while its negative effects are well known, including heart disease and lung and throat cancer. Potentially, they both affect the same mechanisms, though a meta-analysis showed that they were independent [53].

Regardless, unless individuals suffer from pre-disposition to PD, it would appear taking caffeine as a preventative measure would be unwise. Taking it to delay the onset of progression appears to be a little more effective, with one study demonstrating that neither coffee nor tea (nor smoking) delayed the progression from minimal to moderate disability with postural reflex impairment, as assessed by the Hoehn and Yahr scale [54], and another more recent study saying the same [55].

Interestingly, another study using the A2aR antagonist istradefylline in a randomized trial showed that even in patients with the most advanced PD, it reduced the time spent in the dyskinesia 'off' state, though it did not reduce severity when the symptoms set in [56]. As caffeine acts primarily by the same mechanism, some effects on disease progression might be expected. Notably, another study showing that there was no significant change in the Unified Parkinson Disease Rating Scale (UPDRS) score with caffeine found that the activities of daily living (ADL) associated with the disease score did almost significantly improve (p=0.081) [57], as shown in Table 2.

**Table 2:** Comparison of primary outcome measures (need for symptomatic therapy and change in total UPDRS score) and secondary outcome measures (change in motor UPDRS score and change in ADL-UDPRS score) for those in the lowest quartile for caffeine use versus those in the highest quartile for caffeine use Although there was no change in requirement for therapy or general UPDRS score, those associated with activities of daily living were almost significant in both the FS1 and FS-TOO studies analyzed (p=0.081 and p=0.231, respectively). ADL - activities of daily living; UPDRS - Unified Parkinson Disease Rating Scale; FS1 - first futility trial; FS-TOO - second futility trial Table adapted from [57], and permission was obtained from the original publisher to reproduce the content.

	Need for symptomatic therapy?		Mean change in total UPDRS (95% CI)	Mean change in motor UPDRS (95% CI)	Mean change in ADL-UPDRS (95% CI)
FS1					
	No	Yes			
Lowest quartile (no. subjects, %)	23 (52.3)	21 (47.7)	8.1 (5.2-11.1)	4.6 (2.4-6.7)	3.0 (1.9-4.1)
Highest quartile (no. subjects, %)	24 (49.0)	25 (51.0)	6.0 (3.5-8.6)	3.9 (2.1-5.7)	1.7 (0.8-2.7)
P	0.751		0.279	0.626	0.081
FS-TOO					
	No	Yes			
Lowest quartile (no. subjects, %)	23 (46.9)	26 (53.1)	7.1 (4.7-9.5)	4.8 (2.9-6.7)	2.0 (1.3-2.8)
Highest quartile (no. subjects, %)	25 (47.2)	28 (52.8)	8.5 (6.1-11.0)	5.3 (3.4-7.2)	2.7 (1.9-3.4)
P	0.981		0.418	0.729	0.231

Therefore, it is probable that caffeine is having some effect on patients with pre-existing PD, but it is not as beneficial as if taken prior to disease onset. This may reflect the distinct actions of caffeine to act as an anti-inflammatory that reduces the destruction of dopaminergic neurons, which are mostly (>70-90%) destroyed by the time of disease onset, and its ability to inhibit GABA signaling. The latter but not the former could still be effective in PD sufferers, thus explaining why difficulty with day-to-day activities might be alleviated but not the progression of the disease.

Further research

Although this area is widely studied and the data fairly conclusive, one problem that is rarely corrected in human studies is the use of coffee and tea in place of raw caffeine. As some of the data suggest the contaminants may have a significant effect, it would be interesting to see a randomized control trial of the use of caffeine pills as a preventative measure - also helping to regulate the dose. The target group would best include individuals predisposed to PD or individuals with substantial SNpc deterioration prior to the onset of symptoms, as these are the people who would most benefit.

In mouse models, it would be interesting to see the effects of caffeine and MPTP on non-inbred strains and an inter-laboratory study using multiple strains.

## Conclusions

Parkinson's disease is an increasing problem within aging populations, and caffeine consumption has been shown near consistently in human and mouse studies to delay the onset of progression in a dose-dependent manner. However, at least in humans, it is much less effective at attenuating the progression of the disease. The results suggest that this action occurs primarily via the competitive inhibition of the A2aRs in the hematopoietic and potentially neuronal cells that results in reduced microglial cell activation and prevention of the inflammatory environment. However, whether caffeine should be used as a preventative measure, despite its efficacy, will depend on the individual due to the increased risk of developing cardiovascular disease, perhaps limiting it to those with a genetic predisposition to PD.
